# Uncovering Key Metabolic Determinants of the Drug Interactions Between Trimethoprim and Erythromycin in *Escherichia coli*

**DOI:** 10.3389/fmicb.2021.760017

**Published:** 2021-10-20

**Authors:** Qin Qi, S. Andreas Angermayr, Tobias Bollenbach

**Affiliations:** ^1^Institute of Science and Technology Austria, Klosterneuburg, Austria; ^2^Institute for Biological Physics, University of Cologne, Cologne, Germany; ^3^Center for Data and Simulation Science, University of Cologne, Cologne, Germany

**Keywords:** transcriptomics, antibiotic interactions, sulfate reduction, trimethoprim (TMP), erythromycin (ERY), *E. coli*

## Abstract

Understanding interactions between antibiotics used in combination is an important theme in microbiology. Using the interactions between the antifolate drug trimethoprim and the ribosome-targeting antibiotic erythromycin in *Escherichia coli* as a model, we applied a transcriptomic approach for dissecting interactions between two antibiotics with different modes of action. When trimethoprim and erythromycin were combined, the transcriptional response of genes from the sulfate reduction pathway deviated from the dominant effect of trimethoprim on the transcriptome. We successfully altered the drug interaction from additivity to suppression by increasing the sulfate level in the growth environment and identified sulfate reduction as an important metabolic determinant that shapes the interaction between the two drugs. Our work highlights the potential of using prioritization of gene expression patterns as a tool for identifying key metabolic determinants that shape drug-drug interactions. We further demonstrated that the sigma factor-binding protein gene *crl* shapes the interactions between the two antibiotics, which provides a rare example of how naturally occurring variations between strains of the same bacterial species can sometimes generate very different drug interactions.

## Introduction

**FIGURE 1:**
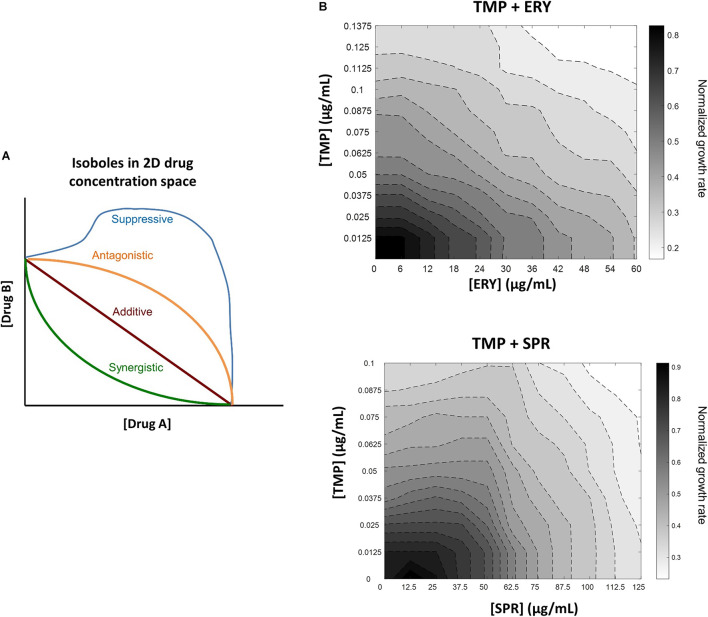
In contrast to another ribosome-targeting antibiotic spiramycin (SPR), erythromycin (ERY) does not suppress trimethoprim (TMP) when tested against a *crl-*deficient MG1655 strain. **(A)** Schematic of different drug-drug interaction isoboles in a two-dimensional drug concentration space. Loewe additivity of drug-drug interactions is used for quantifying effects of drug combinations. Lines of constant inhibition (isoboles) in the 2D drug concentration space represent combinations of drugs A and B that result in same normalized growth rate. The four types of interactions shown are synergistic (green), additive (red), antagonistic (orange) and suppressive (blue). **(B)** TMP and ERY show almost additive interaction against *crl-*deficient *E. coli* MG1655. Isoboles in grayscale represent exponential growth rates normalized to that of the no-antibiotic treatment control in 2D concentration space. When tested against a *crl-*deficient *E. coli* MG1655 strain, ERY and TMP show an additive interaction. In contrast, SPR exerted a mildly suppressive effect on TMP under the same experimental conditions.

A growing volume of microbiology research has been dedicated to characterizing drug-drug interactions in combination antibiotic treatment, because understanding how and why antibiotics interact with each other has the potential to help clinicians improve treatment efficacy, reduce dosage-related side effects and slow the emergence of antibiotic resistance (Tamma et al., 2012; Worthington and Melander, 2013; Mitosch and Bollenbach, 2014; Bollenbach, 2015; Wood, 2016). The use of high-throughput assays for measuring bacterial growth rate has accelerated the identification and characterization of antibiotic interactions (Yeh et al., 2006; Wood et al., 2012; Ocampo et al., 2014; Chevereau and Bollenbach, 2015; Zhou et al., 2015; Nguyen et al., 2016). Pairwise interactions between antibiotics can be classified as additive, synergistic or antagonistic (Figure 1A) based on whether their combined effects on bacterial growth inhibition are equal to, greater than or less than those of their individual effects, respectively (Loewe, 1928, 1953; Bliss, 1939; Yeh et al., 2006; Foucquier and Guedj, 2015). Suppression is a special type of antagonism in which the combined growth inhibitory effect is weaker than that of one antibiotic or both antibiotics alone (Yeh et al., 2006).

Our current understanding of the origins of antibiotic interactions still lacks comprehensive mechanistic underpinnings. There are several well-studied examples that explain why drugs that target the same pathways tend to interact synergistically. For example, trimethoprim and sulfamethoxazole inhibit two steps in the tetrahydrofolic acid synthesis pathway. Their synergistic interaction has been attributed to the drugs’ ability to potentiate each other’s inhibitory effects on key metabolite synthesis within this pathway (Minato et al., 2018). The synergism between two ribosome-targeting antibiotics of the macrolide family, lankacidin and lankamycin, can be explained by their ability to target neighboring sites in the ribosome (Auerbach et al., 2010). For antibiotics that target unrelated pathways, the ability of one drug to affect the uptake or efflux of another has been proposed to be a mechanism that results in drug interactions (Cokol et al., 2011; Brochado et al., 2018). For example, the synergism between streptomycin and penicillin was proposed to be due to the cell membrane-damaging effect of penicillin, which in turn facilitates bacterial cell penetration by streptomycin (Plotz and Davis, 1962). Conversely, antagonism can emerge when one drug limits the proton motive force-dependent uptake of another drug or increases its efflux through efflux pumps, resulting in decreased intracellular concentration of the latter (Brochado et al., 2018).

The suppressive effect of spiramycin, a ribosome-targeting antibiotic, on trimethoprim has been attributed to non-optimal regulation of ribosomal genes, which leads to an imbalance between cellular DNA and protein contents in the presence of trimethoprim-induced DNA stress (Bollenbach et al., 2009). This mechanism also underlies the antagonism between ciprofloxacin and tetracycline and has been proposed to hold more generally for antibiotic pairs comprising a DNA synthesis inhibitor and a protein synthesis inhibitor (Chait et al., 2007; Bollenbach et al., 2009). More broadly, polysaccharide and ATP synthesis in *E. coli* are key cellular processes that shape pairwise drug interactions between six antibiotics with different modes of action; supplementing growth medium with small molecule adjuvants that target these cellular functions alters drug-drug interactions in a predictable way (Chevereau and Bollenbach, 2015).

Erythromycin shares similarities with spiramycin with respect to their inhibitory effect on translation elongation (Kirst and Sides, 1989). Both are macrolide-type ribosome-targeting bacteriostatic antibiotics that bind to the 50S large ribosomal subunit, block nascent polypeptides (Hansen et al., 2002), and trigger the cold shock response in *E. coli* (van Bogelen and Neidhardt, 1990). Erythromycin is expected to suppress trimethoprim. However, the interaction between erythromycin and trimethoprim was experimentally characterized as additive (Ocampo et al., 2014) or predicted to be additive against *E. coli* (Wood et al., 2012). What causes this apparent lack of a suppressive interaction between erythromycin and trimethoprim? To address this question, we applied an RNA-Seq transcriptomics approach to capture the effects of single and combined antibiotic treatments on gene expression in *E. coli*. Gene regulation responses often show conflicts when antibiotics that generate different transcriptional responses are combined. Bacteria often resolve such conflicts by prioritizing gene expression to one of the two drugs or by averaging the gene expression due to both drugs (Bollenbach and Kishony, 2011; Young and Elowitz, 2011). We explored whether this phenomenon could help us identify genetic determinants that shape the interactions between erythromycin and trimethoprim. Notably, our study revealed that when the two antibiotics are combined, the expression of sulfate reduction genes escaped the stronger overall effect of trimethoprim on the transcriptome. This unexpected transcriptomic response underlies the lack of a suppressive interaction in a sulfate-limited growth environment. In addition, we showed that the presence of an intact sigma factor-binding protein gene *crl* also contributes to a suppressive interaction between the two antibiotics. Overall, our findings highlight the roles of gene expression prioritization patterns of an essential metabolic pathway and a key genetic background variation in shaping drug interactions.

## Materials and Methods

### Growth Medium, Antibiotics and Bacterial Strains

LB broth Miller (pH 7.4) was used as the default growth medium throughout this study, and all chemicals were obtained from Sigma-Aldrich (St. Louis, United States). Where required, LB broth was supplemented with 2 mM sodium sulfate. Trimethoprim (TMP) was dissolved in dimethyl sulfoxide, while erythromycin (ERY) and spiramycin (SPR) were dissolved in ethanol to obtain 50 mg/mL stock solutions. The *E. coli* MG1655 strain in this study is a *crl-*deficient strain used in a previous work with a 777 bp IS1 transposable element insertion in the *crl* coding region (Bollenbach et al., 2009). The *E. coli* BW25113 ancestral strain and the *crl* single gene knockout derivative strain of BW25113 are from the Keio collection (Baba et al., 2006). To account for the kanamycin resistance marker that is present in the *crl* knockout strain, BW25113 was transformed with the low copy number pUA66 plasmid (Zaslaver et al., 2006) to generate the kanamycin-resistant BW25113/pUA66 control strain. The IS1 insertion in *crl* of our MG1655 strain and the intact *crl* in the BW25113 parental strain were verified using the primer pair 5′-CAGGAAATCACCGACTGGAT-3′ and 5′-CGACGTCGGTGCTACGTATT-3′ (Freddolino et al., 2012).

### Growth Rate Measurement and Data Analysis

Prior to each kinetic growth rate experiment in 96-well microtiter plates, *E. coli* strains were pre-cultured overnight for approximately 20 h in antibiotic-free LB broth with shaking (225 rpm) at 37°C. On the next day, antibiotic stock solutions for trimethoprim and one of the two macrolides (ERY or SPR) were diluted in LB broth to obtain linear concentration gradients, which were subsequently used to set up two-dimensional antibiotic concentration gradients in clear, flat-bottom 96-well microtiter plates with lids (Thermo Fisher Scientific, United States). The overnight pre-culture was diluted 1:1,000 in 200 μl cultures with and without antibiotics. Six technical replicates of cultures with no antibiotic treatment were included in each microtiter plate as control. The microtiter plates were incubated for 16–18 h at 37°C with continuous shaking on a Synergy H1 Hybrid Multi-Mode Reader (BioTek, United States). Absorbance at the 600 nm wavelength (OD_600_) was measured every 10 min. Only growth curves of sub-lethal antibiotic treatments were retained for further analyses. Growth curves that showed lethal effects associated with high concentrations of single or combination antibiotic treatments were excluded even if an initial period of transient growth can be recorded.

Calculation of exponential growth rates was performed in MATLAB (MathWorks, United States). First, OD_600_ measurements from wells that contained blank LB broth were subtracted from all data points. Blank-corrected absorbance measurements (OD_600_) that lie within the early exponential phase (defined here as 0.02 < OD_600_ < 0.18) were log-transformed. An array of linear regression values within the interval was calculated for each culture using all combinations of six consecutive data points for every bacterial culture. A 60-min sliding window determined the maximum exponential growth rate within this interval. Normalized exponential growth rates of bacterial cultures with antibiotic treatments were calculated by normalizing their maximum exponential growth rate to that of the no-antibiotic treatment control in the same experiment. Each growth rate experiment was performed three times to obtain three independent biological replicates. Arrays of average normalized exponential growth rates in the [0, 1] interval were thus obtained for each experimental condition involving different *E. coli* strains, antibiotic pairs or growth media, which were noise-filtered using the **ordfilter2** function with a round filter. Isoboles representing the same levels of normalized exponential growth rates in the two-dimensional concentration space of antibiotic pairs (isocontours) were visualized using the **contour** function.

### RNA-Seq

Prior to RNA isolation, bacterial cultures were grown in the same conditions as described above in LB medium (Miller). Two biological replicates of total RNA samples were isolated for each antibiotic-treatment and the no-treatment control (Table 1). Eight technical replicates were included for every condition for each biological replicate. After 3 h ± 20 min of growth, exponential phase bacterial cultures (OD_600_ < 0.35) were mixed with RNAprotect Bacteria Reagent (Qiagen, Germany), and total RNA was extracted using the SV Total RNA Isolation System (Promega, United States) according to the manufacturers’ instructions with minor modifications. The RNA integrity numbers (RIN) of all the total RNA samples were assessed using the Agilent RNA 6000 Nano Kit and Agilent 2100 Bioanalyzer (Agilent Technology, United States) and were in the 8.8 ≤ RIN ≤ 10 range.

**TABLE 1:** Sample groups TMP, ERY, TMP + ERY and the no-antibiotic treatment control for RNA-Seq transcriptomic profiling using the *E. coli* MG1655 strain.

Sample group	Description
No-antibiotic control	Control group without antibiotic treatment
TMP	Trimethoprim-treatment resulting in 50% reduction in exponential growth rate (IC_50_); concentration of TMP = 0.0625 μg/mL
ERY	Erythromycin-treatment resulting in 50% reduction in exponential growth rate (IC_50_); concentration of ERY = 42 μg/mL
TMP + ERY	Combination treatment with trimethoprim and erythromycin, both of which were used at their respective IC_50_ concentrations
TMP (0.2 × IC_50_) + ERY (0.6 × IC_50_)	Combination treatment with trimethoprim and erythromycin that resulted in approximately 50% reduction in exponential growth rate relative to the no-antibiotic control group

Illumina high-throughput sequencing was performed by the Vienna Biocenter Core Facility. Ribodepletion of total RNA was carried out using the Ribo-Zero rRNA Removal Kit (Epicenter, United States). mRNA library was generated using the NEBNext Ultra Library Prep Kit for Illumina (NEB, United States). For multiplexing, TruSeq adaptors were ligated to individual samples. Paired-end sequencing was carried on HiSeq 2500 V4 with read length of 125 bp (Illumina, United States).

### Transcriptomic Data Analysis

Raw Illumina reads were trimmed and filtered using the NGS QC Toolkit (Patel and Jain, 2012). The 3′ and 5′ ends of raw reads were trimmed when *Phred* score < 20. Trimmed reads that were shorter than 125 bp were discarded. Next, filtered reads with *Phred* score > 20 throughout 80% of their lengths were retained, while reads with ambiguity in > 2% of all bases were discarded. Illumina adaptors were removed using TrimGalore (Martin, 2011). Using Bowtie 2, the trimmed and filtered reads were mapped to the *E. coli* MG1655 reference genome with gene annotations (genome assembly ASM584v2) from Ensembl Bacteria (Langmead and Salzberg, 2012; Yates et al., 2016). BAM/SAM file manipulations were carried out using SAMtools commands for viewing, sorting and indexing (Li et al., 2009). All BAM (*.bam), BAM index (*.bam.bai) and reference genome files are available on the Dryad repository (see Supplementary Table 1). Gene counts were extracted from the sorted and indexed BAM files using the Python script *HTSeq* (Anders et al., 2014). After excluding genes with no reads, the median sequencing coverage was 683x.

Using the *DESeq2* Bioconductor package (Anders and Huber, 2010; Anders et al., 2013), genes with an average of fewer than 10 reads per sample across all the experimental conditions were discarded. For each gene, the geometric mean for gene counts was calculated across all sample groups, which was applied as the normalization factor for that gene. Within each sample, the median of all normalized gene counts was applied as the size factor for that sample in the second normalization step. For principal component analysis (PCA), logarithmic transformation of the normalized count data was carried out to remove the dependence of the variance on the mean. PCA was performed using default parameters of the **plotPCA** function. The PCA score plot was generated using the 500 most variable genes across all the sample groups in our dataset. Briefly, the covariance matrix computed from the logarithm of normalized count data was decomposed into eigenvectors, which were used as weighting coefficients to calculate the principal component scores (Todorov et al., 2018). Top/bottom gene loading on the PCA were calculated using the Bioconductor package *pcaExplorer* (Marini and Binder, 2019). Heat-map diagrams and dendrograms from the two-way hierarchical clustering analysis of the 100 most variable genes were generated using the **heatmap.2** function. For every gene, the logarithm of normalized gene counts was transformed into *Z*-scores such that their mean is 0 and standard deviation is 1 across all the sample groups.

For differential gene expression analysis, a negative binomial general linear model was fitted for each gene, and the Wald test was applied for significance testing. In samples that were treated with one or two antibiotics, genes are classified as differentially expressed relative to the no-treatment control group if their adjusted *P*-values are less than 0.01 after controlling for false discovery rate using Benjamini-Hochberg correction (Benjamini and Hochberg, 1995). Differentially expressed genes were compiled for five pairwise comparisons: (1) TMP against no-drug control, (2) ERY against no-drug control, (3) TMP + ERY against no-drug control, (4) TMP + ERY against ERY and (5) TMP + ERY against TMP. Venn diagrams were constructed for the differentially expressed genes in pairwise comparisons (1), (2) and (3). Pathway enrichment analysis was performed for pairwise comparison (5) using the GAGE (Generally Applicable Gene-set Enrichment) package (Luo et al., 2009). Enrichment of the assimilatory sulfate reduction KEGG pathway (eco00920) in a wider context of sulfur metabolism was visualized using the Bioconductor package Pathview (Luo and Brouwer, 2013; Kanehisa et al., 2016). Across pairwise comparisons (1), (2) and (3), two-way hierarchical clustering analysis of average log_2_ fold-change for *cys* genes was performed using the heatmap.2 function.

### Quantitative Real-Time PCR

In three biological replicates, *E. coli* MG1655 was incubated at 37°C overnight with shaking in 2 mL antibiotic-free LB medium as three independent cultures. On the next day, saturated pre-cultures were diluted 1:500 in 2 mL fresh LB medium (Miller) containing TMP, ERY + TMP or ERY + TMP + 2 mM sodium sulfate, where ERY and TMP concentrations are 42 and 0.0625 μg/mL, respectively. After approximately 3 h of further incubation with shaking (225 rpm), total RNA was extracted from each bacterial pellet using a phenol-chloroformed based method with QIAzol Lysis Reagent (Qiagen, Germany). After treatment with DNase I solution at 37°C (New England Biolabs), 200 ng total RNA of each sample was added as template for cDNA synthesis using the LunaScript RT SuperMix Kit (New England Biolabs, United States) according to the manufacturer’s instructions.

For qRT-PCR, equal volumes of each cDNA template were amplified using Luna Universal qPCR Master Mix (New England Biolabs, United States) and 0.25 μM gene-specific oligonucleotide primer pairs for the target gene *cysH* (5′-CTGAATGCCAAAGCTGGAAG-3′; 5′-AACGCCGAACTGGA AAAACT-3′), as well as the internal reference genes *rpoB* (5′-GTAAGGCACAGTTCGGTGGT-3′; 5′-ATTTCCTGCAGGG TGTATGC-3′) and *secA* (5′-GGTAGTCGTAACGATCGCA-3′; 5′-TTTCCATCTCCGGTTCCAT-3′). The thermocycler profile on the LightCycle 480 System (Roche, United States) comprised 60 s of initial denaturation at 95°C, followed by 45 cycles of denaturation (15 s at 95°C), extension (30 s at 60°C) and fluorescence signal acquisition. Reactions were carried out in two technical replicates for the three biological replicates. Using the 2^–ΔΔCT^ method, the relative expression levels of *cysH* were normalized to the average expression of *rpoB* and *secA*. Batch effect was accounted for by normalizing *cysH* expression levels in the TMP + ERY and TMP + ERY + 2 mM sulfate groups to those in the TMP-only reference group within each set of biological replicates. Relative expression levels of *cysH* are expressed in log_2_ fold-change. Error bars denote standard error of the mean.

## Results

To characterize the interactions between trimethoprim (TMP) and erythromycin (ERY) against an *E. coli* MG1655 wild-type strain, we set up a two-dimensional concentration gradient of the two antibiotics in LB medium and measured the optical density (600 nm) of bacterial cultures over time using kinetic assays. All experiments were carried out in LB medium at 37°C due to the availability of existing literature that focused on the inhibitory effects of sub-lethal TMP or ERY treatments under similar experimental conditions (Chevereau and Bollenbach, 2015; Giroux et al., 2017; Chen and Wang, 2021). The maximum growth rates of bacteria were quantified during the early exponential phase. The isoboles in the contour plots represent pairwise combinations of antibiotic concentrations that resulted in the same exponential growth rates relative to the no-antibiotic treatment controls. The overall shape of the contours suggests that the interaction between TMP and ERY is almost additive (Figure 1B). We then performed the same experiment for TMP and spiramycin (SPR) and found that SPR has a clearly antagonistic, and even mildly suppressive effect on TMP (Figure 1B), which is in agreement with previous results despite the differences in experimental conditions (Bollenbach et al., 2009). Our results confirmed that ERY and SPR show different pairwise interactions with TMP under the growth conditions in this study.

**FIGURE 2:**
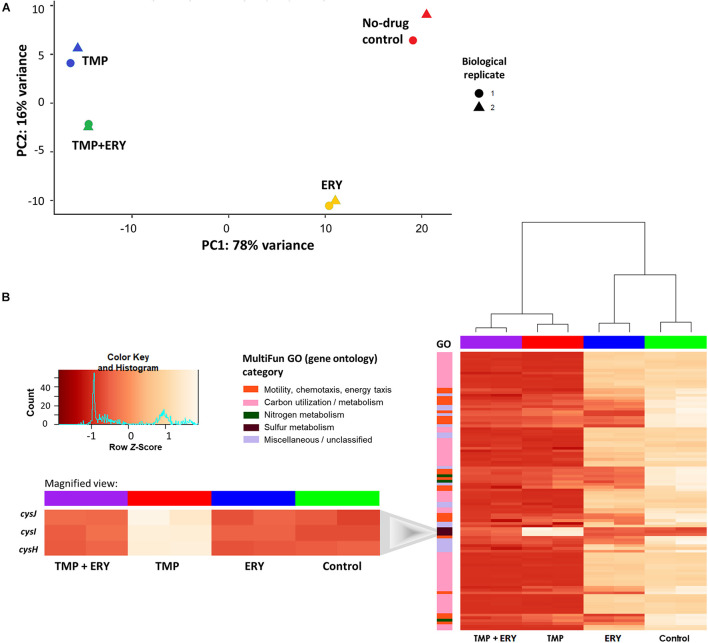
In TMP + ERY combination treatment, TMP exerts a much stronger overall effect on the transcriptomic response than ERY does. **(A)** TMP triggers a different transcriptomic response than ERY but is similar to the combination treatment of TMP + ERY. Principal component 1 (PC1) explains 78% of the total variance in gene expression, while PC2 accounts for 16%. TMP (blue) and ERY (yellow) trigger different transcriptomic responses, and the response to TMP + ERY treatment (green) is more similar to TMP than it is to ERY. **(B)** Except for three sulfur metabolism related genes, TMP and TMP + ERY show a high degree of similarity in gene expression profiles. Normalized gene counts from RNA-Seq were *Z*-score transformed across all sample groups before a two-way hierarchical clustering analysis was performed based on the top 100 genes with the highest variance. The heatmap revealed that the transcriptomic responses for the TMP and TMP + ERY groups were highly similar across four MultiFun gene ontology (GO) categories for 97 genes. The only exception is the sulfur metabolism category in which *cysH* and the *cysIJ* operon do not display TMP-induced upregulation in the TMP + ERY group (inset).

To identify genes and cellular pathways that shape the interaction between TMP and ERY, we obtained the RNA-Seq profiles of exponentially growing cultures treated with single antibiotics at their half-maximal inhibitory concentrations (IC_50_), as well as with a combination of these TMP and ERY concentrations using paired-end Illumina HiSeq (Table 1). Principal component analysis (PCA) was used to dissect how each treatment contributes to the total variance in gene expression and visualize the distribution of sample groups on a PCA score plot (Figure 2A). TMP and ERY triggered distinct transcriptomic responses and formed separate clusters. The position of the TMP + ERY combination treatment group is highly similar to that of TMP on PC1, which explains most of the variance (78%). It is mostly on PC2, which captures 16% of the variance, where the TMP + ERY group differs from the TMP group. Differential gene expression analysis relative to the no-antibiotic control group was performed (Supplementary File 1). The Venn diagram in Supplementary Figure 1A shows that the number of differentially expressed genes (Wald test: *P* < 0.01 after Benjamini-Hochberg correction) was much higher for TMP (2,443 genes) compared to ERY (1,008 genes). Linear regression analysis (Supplementary Figure 1B) revealed a high degree of similarity between the log_2_ fold-changes of differentially expressed genes that are common to the TMP and TMP + ERY groups (coefficient of determination, *R*^2^ = 0.925). Overall, the changes in transcriptomic profiles of the TMP + ERY group are driven by the stronger transcriptomic responses associated with TMP.

Next, we performed a two-way hierarchical clustering analysis to contrast the gene expression profiles of the top 100 genes with the greatest variations across all sample groups. Almost all the genes in the TMP + ERY group displayed gene expression responses that are consistent with those in the TMP group (Figure 2B). The only clear exceptions are the *cysH* gene and *cysIJ* operon from the assimilatory sulfate reduction pathway, which belongs to the sulfur metabolism gene ontology (GO) category. The expression levels of these three genes in the TMP + ERY group resemble those in the ERY and the no-drug control groups. Differential gene expression analysis indicates that TMP led to significant upregulation of *cysH* and *cysIJ* relative to the no-antibiotic control group, while the TMP + ERY and ERY groups showed expression levels that are similar to the control group (Figure 2B inset). In other words, ERY in the TMP + ERY combination treatment lowered the expression of *cysH* and *cysIJ* relative to that in the TMP-treatment group.

**FIGURE 3:**
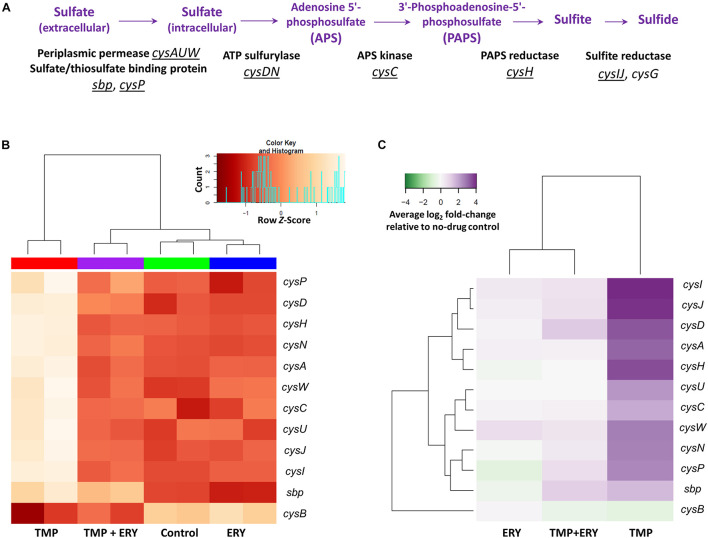
Transcriptomic response of the assimilatory sulfate reduction pathway. **(A)** Genes and operons of the assimilatory sulfate reduction pathway. The underlined genes and operons were included in the two-way hierarchical clustering analysis. *cysG* was excluded from this analysis due to its different regulatory mechanism from all other genes in the sulfate reduction pathway. **(B)** The expression profiles of the sulfate reduction pathway follow the same trend as *cysH* and *cysIJ*. Two-way hierarchical clustering analysis for genes of the sulfate reduction pathway and their transcriptional regulator gene *cysB*. With the exceptions of *cysB* and *sbp*, all the *cys* genes in this pathway generally follow the same gene expression profiles as those of *cysH* and *cysIJ* across all the sample groups. **(C)** Average log_2_ fold-change of sulfate reduction gene expression levels in the TMP, ERY and TMP + ERY groups relative to the no-drug control group.

We then examined the wider implications of the distinct expression patterns for *cysH* and *cysIJ*. Sulfate reduction genes are known to be differentially expressed when *E. coli* is treated with TMP (Sangurdekar et al., 2011; Mitosch et al., 2017). The assimilatory sulfate reduction pathway (Figure 3A) scavenges extracellular sulfate and reduces intracellular sulfate to sulfide *via* several intermediate steps (Sekowska et al., 2000). This pathway is activated by the transcriptional activator CysB and the *N*-acetyl-L-serine inducer (Ostrowski and Kredich, 1989; Neidhardt, 1996). The end-product of the pathway, sulfide, depletes the *N*-acetyl-L-serine inducer *via* the *O*-acetyl-L-serine precursor, and thus exerts negative feedback on the expression of *cys* genes (Neidhardt, 1996). CysB is also negatively regulated by itself and *N*-acetyl-L-serine (Jagura-Burdzy and Hulanicka, 1981; Neidhardt, 1996). We hypothesized that the distinct expression patterns of *cysH* and *cysIJ* in the TMP + ERY group extends to other sulfate reduction genes, because they share the same transcriptional regulatory mechanism.

We performed a separate two-way hierarchical clustering analysis for *cysB* and the sulfate reduction genes of Figure 3A using row *Z*-scores and average log_2_ fold-change relative to the no-drug control. With the exception of *cysB* and the periplasmic sulfate-binding protein gene *sbp*, the expression profile of all the *cys* genes in the sulfate reduction pathway followed the same trend as *cysH* and *cysIJ* (Figures 3B,C). To exclude the possibility that this was a consequence of differences in growth rates across the four sample groups, we compared the TMP (IC_50_) and ERY (IC_50_) groups with an additional TMP + ERY sample group with approximately 50% reduction in exponential growth rate. This was achieved by reducing TMP and ERY concentrations for this combination treatment group. Two-way hierarchical clustering analysis confirmed that the TMP (0.2 × IC_50_) + ERY (0.6 × IC_50_) group clustered with ERY rather than with TMP (Supplementary Figure 3), which is consistent with the trend in Figure 3B. This suggests that the sulfate reduction genes are generally restrained from responding to TMP when ERY is also present.

Treating *E. coli* with TMP in LB medium causes oxidative stress (Giroux et al., 2017), which is expected to compromise sulfate reduction and promote auto-oxidation of sulfide (Dolla et al., 2006). LB medium is also considered to be a sulfate-poor growth medium and contains approximately 0.1–0.15 mM of sulfate (Kertesz, 2000). The upregulation of the sulfate reduction pathway in the presence of TMP is a transcriptomic signature of sulfur limitation and decrease in intracellular sulfide availability (Neidhardt, 1996; Gyaneshwar et al., 2005). Thus, it is consistent with the negative feedback transcription regulation for this pathway that counteracts sulfur limitation (Neidhardt, 1996). Further links between oxidative stress and upregulation of the sulfate reduction pathway had previously been established by transcriptomic studies in which *E. coli* cultures grown in LB medium were treated with oxidizing agents such as chlorine, hydrogen peroxide, paraquat and sodium salicylate (Pomposiello et al., 2001; Wang et al., 2009). These are consistent with the TMP-induced upregulation of assimilatory sulfate reduction genes we observed. However, it is conceivable that other TMP-induced effects on one-carbon metabolism, as well as methionine and *S*-Adenosyl methionine (SAM) synthesis could also have contributed to the observed upregulation of the assimilatory sulfate reduction pathway.

Using the TMP versus no-drug control and TMP + ERY versus TMP pairwise comparisons, we performed gene set analysis on the assimilatory sulfate reduction pathway and neighboring nodes of the sulfur metabolism network (KEGG annotation). ERY in the combination treatment suppressed the upregulation of *cys* sulfate reduction genes when TMP + ERY was compared against TMP (Supplementary Figure 4B). A volcano plot for the TMP + ERY versus TMP comparison also confirmed that *cys* genes were amongst some of the most downregulated genes (Supplementary Figure 5). An intriguing observation that emerged from the pathway analysis was the upregulation of *tauD* and *ssuE*/*ssuD*, whose gene products allow taurine and alkanesulfonates, respectively, to be utilized as alternative sulfur sources under sulfate starvation (van der Ploeg et al., 1996; Eichhorn et al., 1999). The lack of an expected upregulation of the sulfate reduction pathway in the TMP + ERY group implies a transcriptional response to sulfur limitation that results in lower fitness.

**FIGURE 4:**
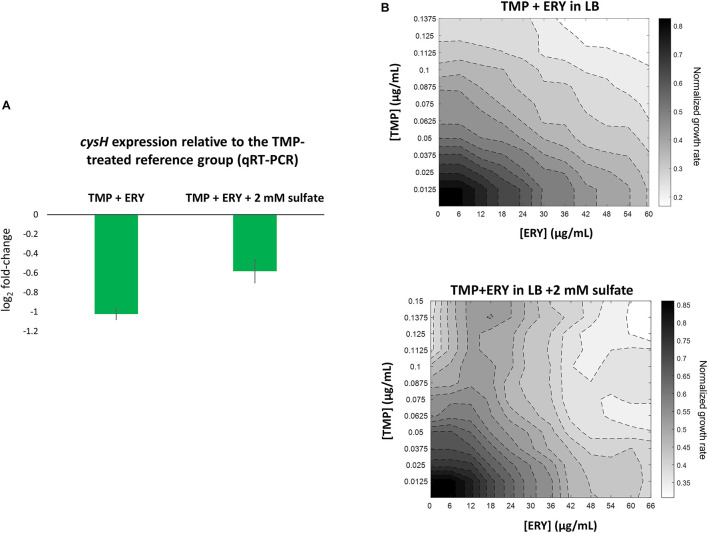
Perturbing the sulfate reduction pathway by adding exogenous sulfate increases *cysH* expression level in TMP + ERY treated MG1655 and changes the TMP + ERY interaction from additive to suppressive. **(A)** qRT-PCR validation of *cysH* expression. Relative to the TMP-treated MG1655 reference group, the TMP + ERY treated group showed an approximately twofold reduction in *cysH* expression, which confirmed the role of ERY in suppressing *cysH* expression. Supplementing the LB growth medium in which the TMP + ERY treated group is grown with 2 mM sulfate caused an increase in average *cysH* relative expression. **(B)** ERY exerts suppression on TMP in growth medium supplemented with sulfate. Isoboles for TMP + ERY drug combinations when tested against the *E. coli* MG1655 strain. Supplementing LB medium with sodium sulfate at 2 mM changed the interaction between TMP and ERY from additive to suppressive. Suppression by ERY on TMP is stronger at higher concentrations of TMP in the presence of exogenous sulfate.

To validate our key observation that ERY in the TMP + ERY combination treatment suppresses the expression of the sulfate reduction pathway, we performed quantitative real-time PCR (qRT-PCR) on *E. coli* MG1655 that had been treated with TMP and TMP + ERY. We focused on the PAPS reductase gene *cysH*, which showed one of the highest variations in expression levels across sample groups (Figure 3B). *cysH* expression was normalized to the average expression of two stably expressed essential genes (according to our RNA-Seq based differential gene expression analysis), *rpoB* (encoding the RNA polymerase β-subunit) and *secA* (encoding the protein translocase subunit SecA) as internal reference genes. As expected, combining ERY with TMP resulted in an approximately twofold reduction in *cysH* expression level relative to the TMP-treated reference group (Figure 4A). Under sulfur limitation, supplementing growth media with sulfate is expected to stimulate the expression of the sulfate reduction pathway (Pasternak et al., 1965; Wheldrake and Pasternak, 1965). To verify this, we added 2 mM sodium sulfate to the LB medium that contained TMP + ERY treated MG1655 and observed an increase in average *cysH* relative expression compared to that without exogenous sulfate. This increase, however, did not fully offset the suppressing effect of ERY on *cysH* expression.

We hypothesized that a transcriptional response to sulfur limitation that is detrimental to fitness in the TMP + ERY group shapes the drug interaction between TMP and ERY. To test this hypothesis, we supplemented LB growth medium with 2 mM sodium sulfate and performed the same growth rate assays as before in TMP + ERY two-dimensional concentration space. Notably, ERY exerts a suppressive effect on TMP in the presence of additional exogenous sulfate, especially at higher TMP concentrations (Figure 4B). The disproportionately large beneficial effect of sulfate on normalized growth rate in the presence of both drugs altered the shape of the isoboles in the 2D drug concentration space (Supplementary Figure 6B).

**FIGURE 5:**
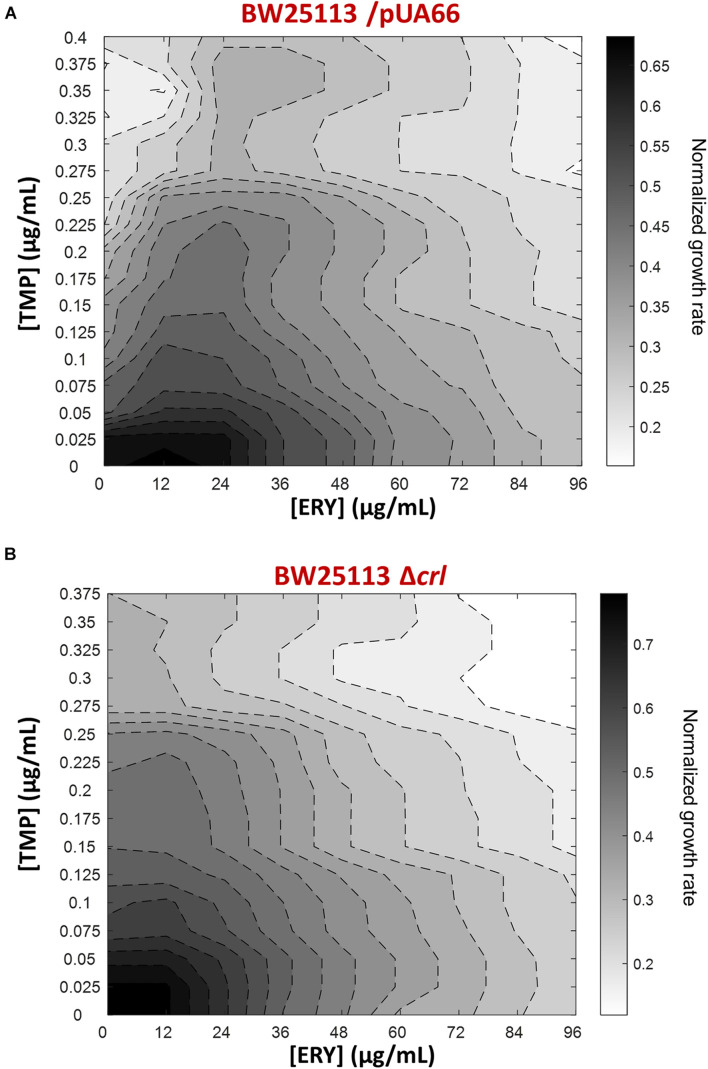
The suppressive effect of ERY on TMP requires the presence of the *crl* gene in BW25113. Isoboles for TMP + ERY drug combinations for the *E. coli* BW25113/pUA66 and BW25113 Δ*crl* strains. Whereas ERY exerts a strong suppressive effect on TMP when tested against the BW25113/pUA66 strain which has an intact *crl* gene **(A)**, the suppression is removed when tested against the BW25113 Δ*crl* strain **(B)**.

To expand our characterization of TMP + ERY interactions to another key reference strain of *E. coli*, we performed additional growth rate assays using the BW25113 strain (Grenier et al., 2014). Although both BW25113 and MG1655 were derived from the *E. coli* K12 ancestral strain, we observed that ERY exerted a pronounced suppressive effect on TMP in the two-dimensional contour plot for BW25113 (Figure 5A). A major difference between BW25113 and the *crl-*deficient MG1655 strain used in this study is that the former carries an intact *crl* gene. Crl is a sigma factor-binding protein and transcription activator that exerts global regulatory effects on the transcriptome by increasing the affinity of RNA polymerase for the principal sigma factor of the stationary phase σ^*S*^ (Typas et al., 2007; Cavaliere and Norel, 2016; Cartagena et al., 2019; Xu et al., 2019). The 86 genes that are controlled by Crl in *E. coli* K-12 form 40% of the RpoS regulon (Santos-Zavaleta et al., 2019). The loss of *crl* can be detected in *E. coli* strains under laboratory settings that favored the inactivation of the general stress response (Faure et al., 2004), as well as in naturally-occurring pathogenic strains of *E. coli* (Provence and Curtiss, 1992; Asadi et al., 2018). We repeated the growth rate experiment using the BW25113 Δ*crl* knockout mutant from the Keio collection to mimic the effects of *crl-*inactivation in the MG1655 strain we used. As expected, deleting *crl* abolished the suppressive drug interaction previously observed (Figure 5B), yielding an antagonistic interaction. Deleting *crl* substantially altered the susceptibility of BW25113 to TMP. The IC_50_ for TMP approximately doubled from 0.10 μg/mL to 0.21 μg/mL, while IC_50_ for ERY only decreased marginally from 48 μg/mL to 42 μg/mL. On the vertical axes of the contour plots, it is evident that the change in shapes of isoboles was mainly driven by the increase in tolerance to TMP in the absence of *crl* (Figure 5). The remaining difference between TMP + ERY interactions in the MG1655 (additive; Figure 1B) and BW25113 Δ*crl* strains (antagonistic; Figure 5B) is most likely to be due to other variations in their genomes.

## Discussion

Applying a transcriptomic approach to identify genetic determinants that affect the interaction between two antibiotics with distinct modes of action is challenging, because the transcriptomic signatures of individual antibiotic treatments are superimposed. We used the interactions between TMP and ERY as a model system, because ERY and SPR show different interactions with TMP (Figure 1B) despite their similarities as ribosome-targeting antibiotics. TMP inhibits folic acid metabolism, depletes key metabolites and induces DNA damage (Sangurdekar et al., 2011; Giroux et al., 2017). Activating global stress responses such as the SOS response and the ppGpp-mediated stringent response has wide-ranging effects on the transcriptome (Courcelle et al., 2001; Khil and Camerini-Otero, 2002; Durfee et al., 2008; Traxler et al., 2008). Despite this, the transcriptomic response of the sulfate reduction pathway in the TMP + ERY group prioritized to that of ERY rather than to TMP (Figure 3 and Supplementary Figure 3). By supplementing the culture medium with exogenous sulfate and perturbing the affected pathway (Figure 4A), we successfully changed the additive interaction between TMP and ERY to a suppressive interaction (Figure 4B). These results demonstrate that analyzing prioritization patterns of conflicting transcriptomic responses can be a useful tool for dissecting drug interaction mechanisms.

Given the disproportionately large beneficial effect of sulfate addition on relative exponential growth rates when the two drugs were combined, we argue that the suppressing effect of *cys* upregulation by ERY is a transcriptional response that does not maximize fitness. The sulfate reduction pathway is a subset of sulfur metabolism, which is subject to strong negative feedback control, because L-cysteine is one of the most toxic amino acids at high concentrations (Datta, 1967; Avcilar-Kucukgoze et al., 2016). In addition to the ability of sulfide to deplete *O*-acetyl-L-serine (precursor to the *N*-acetyl-L-serine inducer), negative feedback is also mediated by the inhibitory effect of L-cysteine on the conversion of serine to *O*-acetyl-L-serine (Neidhardt, 1996). TMP upregulated *cys* genes of the sulfate reduction pathway and slightly downregulated *cysB* (Figures 3B,C), and these changes are consistent with the transcriptional regulatory mechanism of this pathway in response to sulfur limitation. The sulfate binding protein gene *sbp* is the only sulfate reduction gene that showed similar expression levels in both the TMP and TMP + ERY groups (Figure 4B), but *sbp* is known to be activated more specifically by sulfate starvation (van der Ploeg et al., 1996). Given how tightly sulfur metabolism is regulated, it is surprising that the *cys* genes displayed an unusual transcriptional response in TMP + ERY (Figures 3B, 4A). As a ribosome-targeting antibiotic, ERY is not expected to directly affect the intra- and extracellular availability of metabolites for sulfur assimilation. More metabolomic insights into the regulation of the *cys* genes in response to combination treatment may reveal why TMP + ERY treated cells displayed a transcriptional response that is unlikely to be effective in counteracting the effects of TMP in terms of oxidative stress and sulfate limitation.

We also demonstrated that the sigma factor-binding protein gene *crl* plays a significant role in determining the interaction between TMP and ERY against *E. coli*. This finding lends support to the growing recognition that interactions between the same combinations of antibiotics can sometimes differ between bacterial species, as well as between different strains of the same species (Brochado et al., 2018). For example, the interaction between nalidixic acid and tetracycline against several multidrug resistant clones of *Acinetobacter baumannii* and *E. coli* is synergistic, while their interactions in susceptible clones of the same species are not (Gaurav et al., 2021). A previous study showed that drug interactions between antibiotics representing six main modes of action are surprisingly robust against most single gene deletions in *E. coli*, and that only a very small minority of single gene knockout strains produced qualitative changes in drug interactions (Chevereau and Bollenbach, 2015). Therefore, *crl* represents a rare example of single gene deletions that caused such shifts in drug interactions. While it is not clear if there are any interactions between the Crl-RpoS regulon and the assimilatory sulfate reduction pathway, our findings hint at the possibility that the effects of specific gene expression patterns on drug interactions may be contingent on the presence or absence of other genes such as transcriptional regulators. Additional future efforts could also be directed toward systematic discovery of specific genetic contexts in which alterations in drug interactions arise due to variations between strains, including the gain of antimicrobial resistance genes and loss of transcriptional regulators.

In conclusion, drug interaction is a complex phenotype that is determined by antibiotic modes of action (Cokol et al., 2011; Ocampo et al., 2014; Chevereau and Bollenbach, 2015), gene expression patterns (Bollenbach et al., 2009; Bollenbach and Kishony, 2011), genetic variations across species and strains (Chevereau and Bollenbach, 2015; Brochado et al., 2018; Gaurav et al., 2021). Our study exemplifies that metabolite abundance in the growth environment and gene prioritization patterns can also affect drug-drug interactions. It is hoped that our work will pave the way for future studies that employ a combination of transcriptomic and complementary techniques to dissect drug-drug interactions.

## Data Availability Statement

The datasets presented in this study can be found in online repositories. The names of the repository/repositories and accession number(s) can be found at: https://doi.org/10.5061/dryad.bk3j9kdcn.

## Author Contributions

QQ and TB conceived the study. SAA and QQ designed, performed, and analyzed the experiments. QQ performed the bioinformatic analyses. TB supervised and managed the project. All authors contributed to the original draft and edited subsequent versions of the manuscript.

## Conflict of Interest

The authors declare that the research was conducted in the absence of any commercial or financial relationships that could be construed as a potential conflict of interest.

## Publisher’s Note

All claims expressed in this article are solely those of the authors and do not necessarily represent those of their affiliated organizations, or those of the publisher, the editors and the reviewers. Any product that may be evaluated in this article, or claim that may be made by its manufacturer, is not guaranteed or endorsed by the publisher.

## Supplementary Material

Supplementary Figure 1 **(A)** Venn diagram for differentially expressed genes in TMP, ERY and TMP + ERY relative to the no-drug control group. **(B)** Linear regression of log_2_ fold-changes for differentially expressed genes that are common to both the TMP and TMP + ERY groups.

Supplementary Figure 2 **(A)** Top and bottom gene loadings on principal components 1 and 2. **(B)** Scree plot for the principal component analysis.

Supplementary Figure 3 Two-way hierarchical clustering of *Z*-score transformed gene expression levels of the sulfate reduction pathway using sample groups with the same 50% reduction in exponential growth rate.

Supplementary Figure 4 Gene set analysis for the assimilatory sulfate reduction pathway and neighboring nodes (KEGG annotation).

Supplementary Figure 5 Volcano plot for the TMP + ERY versus TMP pairwise comparison.

Supplementary Figure 6 **(A)** Normalized growth rates of TMP + ERY treated MG1655 with and without sulfate supplementation. **(B)** Percentage increase in normalized growth rate with 2 mM sulfate supplementation relative to that in LB without exogenous sulfate.

Supplementary Table 1 Accession numbers of BAM and reference genome files for the RNA-Seq transcriptomic dataset in Dryad repository (doi: 10.5061/dryad.bk3j9kdcn).

Supplementary File 1 Genome-wide differential gene expression in the TMP, ERY and TMP + ERY groups relative to the no-antibiotic control group; as well as in TMP + ERY relative to the ERY and TMP single-antibiotic reference groups.

## References

[B1] Anders S. Huber W. (2010). Differential expression analysis for sequence count data. *Genome Biol.* 11:R106. 10.1186/gb-2010-11-10-r106 20979621 PMC3218662

[B2] Anders S. McCarthy D. J. Chen Y. Okoniewski M. Smyth G. K. Huber W. (2013). Count-based differential expression analysis of RNA sequencing data using R and Bioconductor. *Nat. Protoc.* 8 1765–1786. 23975260 10.1038/nprot.2013.099

[B3] Anders S. Pyl P. T. Huber W. (2014). HTSeq — A python framework to work with high-throughput sequencing data. *bioRxiv* [Preprint]. 10.1093/bioinformatics/btu638 25260700 PMC4287950

[B4] Asadi A. Zahraei Salehi T. Jamshidian M. Ghanbarpour R. (2018). ECOR phylotyping and determination of virulence genes in *Escherichia coli* isolates from pathological conditions of broiler chickens in poultry slaughter-houses of southeast of Iran. *Vet. Res. Forum* 9 211–216. 30357106 10.30466/vrf.2018.30827 PMC6198163

[B5] Auerbach T. Mermershtain I. Davidovich C. Bashan A. Belousoff M. Wekselman I. (2010). The structure of ribosome-lankacidin complex reveals ribosomal sites for synergistic antibiotics. *Proc. Natl. Acad. Sci. U.S.A.* 107 1983–1988. 10.1073/pnas.0914100107 20080686 PMC2804743

[B6] Avcilar-Kucukgoze I. Bartholomäus A. Cordero Varela J. A. Kaml R. F. Neubauer P. Budisa N. (2016). Discharging tRNAs: a tug of war between translation and detoxification in *Escherichia coli*. *Nucleic Acids Res.* 44 8324–8334. 10.1093/nar/gkw697 27507888 PMC5041488

[B7] Baba T. Ara T. Hasegawa M. Takai Y. Okumura Y. Baba M. (2006). Construction of *Escherichia coli* K-12 in-frame, single-gene knockout mutants: the Keio collection. *Mol. Syst. Biol.* 2:2006.0008. 10.1038/msb4100050 16738554 PMC1681482

[B8] Benjamini Y. Hochberg Y. (1995). Controlling the false discovery rate: a practical and powerful approach to multiple testing. *J. R. Stat. Soc. Lond. B Methodol.* 57 289–300. 10.1111/j.2517-6161.1995.tb02031.x

[B9] Bliss C. (1939). The toxicity of poisons applied jointly. *Ann. Appl. Biol.* 26 585–615. 10.1111/j.1744-7348.1939.tb06990.x

[B10] Bollenbach T. Quan S. Chait R. Kishony R. (2009). Nonoptimal microbial response to antibiotics underlies suppressive drug interactions. *Cell* 139 707–718. 10.1016/j.cell.2009.10.025 19914165 PMC2838386

[B11] Bollenbach T. Kishony R. (2011). Resolution of gene regulatory conflicts caused by combinations of antibiotics. *Mol. Cell* 42 413–425. 10.1016/j.molcel.2011.04.016 21596308 PMC3143497

[B12] Bollenbach T. (2015). Antimicrobial interactions: mechanisms and implications for drug discovery and resistance evolution. *Curr. Opin. Microbiol.* 27 1–9. 10.1016/j.mib.2015.05.008 26042389

[B13] Brochado A. R. Telzerow A. Bobonis J. Banzhaf M. Mateus A. Selkrig J. (2018). Species-specific activity of antibacterial drug combinations. *Nature* 559 259–263. 10.1038/s41586-018-0278-9 29973719 PMC6219701

[B14] Cartagena A. J. Banta A. B. Sathyan N. Ross W. Gourse R. L. Campbell E. A. (2019). Structural basis for transcription activation by Crl through tethering of σ. *Proc. Natl. Acad. Sci. U.S.A.* 116 18923–18927. 10.1073/pnas.1910827116 31484766 PMC6754549

[B15] Cavaliere P. Norel F. (2016). Recent advances in the characterization of Crl, the unconventional activator of the stress sigma factor σ^S^/RpoS. *Biomol. Concepts* 7 197–204. 10.1515/bmc-2016-0006 27180360

[B16] Chait R. Craney A. Kishony R. (2007). Antibiotic interactions that select against resistance. *Nature* 446 668–671. 10.1038/nature05685 17410176

[B17] Chen Z. Wang H. (2021). Antibiotic toxicity profiles of *Escherichia coli* Strains Lacking DNA Methyltransferases. *ACS Omega* 6 7834–7840. 10.1021/acsomega.1c00378 33778295 PMC7992158

[B18] Chevereau G. Bollenbach T. (2015). Systematic discovery of drug interaction mechanisms. *Mol. Syst. Biol.* 11:807. 10.15252/msb.20156098 25924924 PMC4422561

[B19] Cokol M. Chua H. N. Tasan M. Mutlu B. Weinstein Z. B. Suzuki Y. (2011). Systematic exploration of synergistic drug pairs. *Mol. Syst. Biol.* 7:544. 10.1038/msb.2011.71 22068327 PMC3261710

[B20] Courcelle J. Khodursky A. Peter B. Brown P. O. Hanawalt P. C. (2001). Comparative gene expression profiles following UV exposure in wild-type and SOS-deficient *Escherichia coli*. *Genetics* 158 41–64. 10.1093/genetics/158.1.41 11333217 PMC1461638

[B21] Datta P. (1967). Regulation of homoserine biosynthesis by L-cysteine, a terminal metabolite of a linked pathway. *Proc. Natl. Acad. Sci. U.S.A.* 58 635–641. 10.1073/pnas.58.2.635 4860755 PMC335682

[B22] Dolla A. Fournier M. Dermoun Z. (2006). Oxygen defense in sulfate-reducing bacteria. *J. Biotechnol.* 126 87–100. 10.1016/j.jbiotec.2006.03.041 16713001

[B23] Durfee T. Hansen A. M. Zhi H. Blattner F. R. Jin D. J. (2008). Transcription profiling of the stringent response in *Escherichia coli*. *J. Bacteriol.* 190 1084–1096. 10.1128/JB.01092-07 18039766 PMC2223561

[B24] Eichhorn E. van der Ploeg J. R. Leisinger T. (1999). Characterization of a two-component alkanesulfonate monooxygenase from *Escherichia coli*. *J. Biol. Chem.* 274 26639–26646. 10.1074/jbc.274.38.26639 10480865

[B25] Faure D. Frederick R. Włoch D. Portier P. Blot M. Adams J. (2004). Genomic changes arising in long-term stab cultures of *Escherichia coli*. *J. Bacteriol.* 186 6437–6442. 10.1128/JB.186.19.6437-6442.2004 15375124 PMC516597

[B26] Foucquier J. Guedj M. (2015). Analysis of drug combinations: current methodological landscape. *Pharmacol. Res. Perspect.* 3:e00149. 10.1002/prp2.149 26171228 PMC4492765

[B27] Freddolino P. L. Amini S. Tavazoie S. (2012). Newly identified genetic variations in common *Escherichia coli* MG1655 stock cultures. *J. Bacteriol.* 194 303–306. 10.1128/JB.06087-11 22081388 PMC3256642

[B28] Gaurav A. Gupta V. Shrivastava S. K. Pathania R. (2021). Mechanistic insights into synergy between nalidixic acid and tetracycline against clinical isolates of *Acinetobacter baumannii* and *Escherichia coli*. *Commun. Biol.* 4:542. 10.1038/s42003-021-02074-5 33972678 PMC8110569

[B29] Giroux X. Su W. L. Bredeche M. F. Matic I. (2017). Maladaptive DNA repair is the ultimate contributor to the death of trimethoprim-treated cells under aerobic and anaerobic conditions. *Proc. Natl. Acad. Sci. U.S.A.* 114 11512–11517. 10.1073/pnas.1706236114 29073080 PMC5664507

[B30] Grenier F. Matteau D. Baby V. Rodrigue S. (2014). Complete genome sequence of *Escherichia coli* BW25113. *Genome Announc.* 2:e01038-14. 10.1128/genomeA.01038-14 25323716 PMC4200154

[B31] Gyaneshwar P. Paliy O. McAuliffe J. Popham D. L. Jordan M. I. Kustu S. (2005). Sulfur and nitrogen limitation in *Escherichia coli* K-12: specific homeostatic responses. *J. Bacteriol.* 187 1074–1090. 10.1128/JB.187.3.1074-1090.2005 15659685 PMC545709

[B32] Hansen J. L. Ippolito J. A. Ban N. Nissen P. Moore P. B. Steitz T. A. (2002). The structures of four macrolide antibiotics bound to the large ribosomal subunit. *Mol. Cell* 10 117–128. 10.1016/S1097-2765(02)00570-1 12150912

[B33] Jagura-Burdzy G. Hulanicka D. (1981). Use of gene fusions to study expression of *cysB*, the regulatory gene of the cysteine regulon. *J Bacteriol* 147 744–751. 10.1128/jb.147.3.744-751.1981 6792186 PMC216109

[B34] Kanehisa M. Sato Y. Kawashima M. Furumichi M. Tanabe M. (2016). KEGG as a reference resource for gene and protein annotation. *Nucleic Acids Res.* 44 D457–D462. 10.1093/nar/gkv1070 26476454 PMC4702792

[B35] Kertesz M. A. (2000). Riding the sulfur cycle–metabolism of sulfonates and sulfate esters in Gram-negative bacteria. *FEMS Microbiol. Rev.* 24 135–175. 10.1016/S0168-6445(99)00033-9 10717312

[B36] Khil P. P. Camerini-Otero R. D. (2002). Over 1000 genes are involved in the DNA damage response of *Escherichia coli*. *Mol. Microbiol.* 44 89–105. 10.1046/j.1365-2958.2002.02878.x 11967071

[B37] Kirst H. A. Sides G. D. (1989). New directions for macrolide antibiotics: pharmacokinetics and clinical efficacy. *Antimicrob. Agents Chemother.* 33 1419–1422. 10.1128/AAC.33.9.1419 2684005 PMC172676

[B38] Langmead B. Salzberg S. L. (2012). Fast gapped-read alignment with Bowtie 2. *Nat. Methods* 9 357–359. 10.1038/nmeth.1923 22388286 PMC3322381

[B39] Li H. Handsaker B. Wysoker A. Fennell T. Ruan J. Homer N. (2009). The sequence alignment/map format and SAMtools. *Bioinformatics* 25 2078–2079. 10.1093/bioinformatics/btp352 19505943 PMC2723002

[B40] Loewe S. (1928). Die quantitativen probleme der pharmakologie. *Ergeb. Physiol.* 27 47–187. 10.1007/BF02322290

[B41] Loewe S. (1953). The problem of synergism and antagonism of combined drugs. *Arzneimittelforschung* 3 285–290. 13081480

[B42] Luo W. Brouwer C. (2013). Pathview: an R/Bioconductor package for pathway-based data integration and visualization. *Bioinformatics* 29 1830–1831. 10.1093/bioinformatics/btt285 23740750 PMC3702256

[B43] Luo W. Friedman M. S. Shedden K. Hankenson K. D. Woolf P. J. (2009). GAGE: generally applicable gene set enrichment for pathway analysis. *BMC Bioinformatics* 10:161. 10.1186/1471-2105-10-161 19473525 PMC2696452

[B44] Marini F. Binder H. (2019). pcaExplorer: an R/Bioconductor package for interacting with RNA-seq principal components. *BMC Bioinformatics* 20:331. 10.1186/s12859-019-2879-1 31195976 PMC6567655

[B45] Martin M. (2011). CutAdapt removes adapter sequences from high-throughput sequencing reads. *EMBnet.J.* 17 10–12. 10.14806/ej.17.1.200

[B46] Minato Y. Dawadi S. Kordus S. L. Sivanandam A. Aldrich C. C. Baughn A. D. (2018). Mutual potentiation drives synergy between trimethoprim and sulfamethoxazole. *Nat. Commun.* 9:1003. 10.1038/s41467-018-03447-x 29520101 PMC5843663

[B47] Mitosch K. Bollenbach T. (2014). Bacterial responses to antibiotics and their combinations. *Environ. Microbiol. Rep.* 6 545–557. 10.1111/1758-2229.12190 25756107

[B48] Mitosch K. Rieckh G. Bollenbach T. (2017). Noisy response to antibiotic stress predicts subsequent single-cell survival in an acidic environment. *Cell Syst.* 4 393–403.e5. 10.1016/j.cels.2017.03.001 28342718

[B49] Neidhardt F. C. (1996). *Escherichia coli* and *Salmonella*: Cellular and Molecular Biology 2nd Edn. Washington, DC: ASM Press.

[B50] Nguyen C. Zhou A. Khan A. Miller J. H. Yeh P. (2016). Pairwise antibiotic interactions in *Escherichia coli*: triclosan, rifampicin and aztreonam with nine other classes of antibiotics. *J. Antibiot. (Tokyo)* 69 791–797. 10.1038/ja.2016.26 26956793

[B51] Ocampo P. S. Lázár V. Papp B. Arnoldini M. Abel zur Wiesch P. Busa-Fekete R. (2014). Antagonism between bacteriostatic and bactericidal antibiotics is prevalent. *Antimicrob. Agents Chemother.* 58 4573–4582. 10.1128/AAC.02463-14 24867991 PMC4135978

[B52] Ostrowski J. Kredich N. M. (1989). Molecular characterization of the *cysJIH* promoters of *Salmonella typhimurium* and *Escherichia coli*: regulation by *cysB* protein and N-acetyl-L-serine. *J. Bacteriol.* 171 130–140. 10.1128/jb.171.1.130-140.1989 2701932 PMC209565

[B53] Pasternak C. A. Ellis R. J. Jones-Mortimer M. C. Crichton C. E. (1965). The control of sulphate reduction in bacteria. *Biochem. J.* 96 270–275. 10.1042/bj0960270 14343143 PMC1206932

[B54] Patel R. K. Jain M. (2012). NGS QC toolkit: a toolkit for quality control of next generation sequencing data. *PLoS One* 7:e30619. 10.1371/journal.pone.0030619 22312429 PMC3270013

[B55] Plotz P. H. Davis B. D. (1962). Synergism between streptomycin and penicillin: a proposed mechanism. *Science* 135 1067–1068. 10.1126/science.135.3508.1067 14487239

[B56] Pomposiello P. J. Bennik M. H. Demple B. (2001). Genome-wide transcriptional profiling of the *Escherichia coli* responses to superoxide stress and sodium salicylate. *J. Bacteriol.* 183 3890–3902. 10.1128/JB.183.13.3890-3902.2001 11395452 PMC95271

[B57] Provence D. L. Curtiss R. (1992). Role of *crl* in avian pathogenic *Escherichia coli*: a knockout mutation of *crl* does not affect hemagglutination activity, fibronectin binding, or curli production. *Infect. Immun.* 60 4460–4467. 10.1128/iai.60.11.4460-4467.1992 1398960 PMC258189

[B58] Sangurdekar D. P. Zhang Z. Khodursky A. B. (2011). The association of DNA damage response and nucleotide level modulation with the antibacterial mechanism of the anti-folate drug trimethoprim. *BMC Genomics* 12:583. 10.1186/1471-2164-12-583 22122981 PMC3258297

[B59] Santos-Zavaleta A. Pérez-Rueda E. Sánchez-Pérez M. Velázquez-Ramírez D. A. Collado-Vides J. (2019). Tracing the phylogenetic history of the Crl regulon through the Bacteria and Archaea genomes. *BMC Genomics* 20:299. 10.1186/s12864-019-5619-z 30991941 PMC6469107

[B60] Sekowska A. Kung H. F. Danchin A. (2000). Sulfur metabolism in *Escherichia coli* and related bacteria: facts and fiction. *J. Mol. Microbiol. Biotechnol.* 2 145–177. 10939241

[B61] Tamma P. D. Cosgrove S. E. Maragakis L. L. (2012). Combination therapy for treatment of infections with Gram-negative bacteria. *Clin. Microbiol. Rev.* 25 450–470. 10.1128/CMR.05041-11 22763634 PMC3416487

[B62] Todorov H. Fournier D. Gerber S. (2018). Principal components analysis: theory and application to gene expression data analysis. *Genom. Computat. Biol.* 4:e100041. 10.18547/gcb.2018.vol4.iss2.e100041

[B63] Traxler M. F. Summers S. M. Nguyen H. T. Zacharia V. M. Hightower G. A. Smith J. T. (2008). The global, ppGpp-mediated stringent response to amino acid starvation in *Escherichia coli*. *Mol. Microbiol.* 68 1128–1148. 10.1111/j.1365-2958.2008.06229.x 18430135 PMC3719176

[B64] Typas A. Barembruch C. Possling A. Hengge R. (2007). Stationary phase reorganisation of the *Escherichia coli* transcription machinery by Crl protein, a fine-tuner of σs activity and levels. *EMBO J.* 26 1569–1578. 10.1038/sj.emboj.7601629 17332743 PMC1829388

[B65] van Bogelen R. A. Neidhardt F. C. (1990). Ribosomes as sensors of heat and cold shock in *Escherichia coli*. *Proc. Natl. Acad. Sci. U.S.A.* 87 5589–5593. 10.1073/pnas.87.15.5589 2198567 PMC54372

[B66] van der Ploeg J. R. Weiss M. A. Saller E. Nashimoto H. Saito N. Kertesz M. A. (1996). Identification of sulfate starvation-regulated genes in *Escherichia coli*: a gene cluster involved in the utilization of taurine as a sulfur source. *J. Bacteriol.* 178 5438–5446. 10.1128/jb.178.18.5438-5446.1996 8808933 PMC178364

[B67] Wheldrake J. F. Pasternak C. A. (1965). The control of sulphate activation in bacteria. *Biochem. J.* 96 276–280. 10.1042/bj0960276 14343144 PMC1206933

[B68] Wang S. Deng K. Zaremba S. Deng X. Lin C. Wang Q. (2009). Transcriptomic response of *Escherichia coli* O157:H7 to oxidative stress. *Appl. Environ. Microbiol.* 75 6110–6123. 10.1128/AEM.00914-09 19666735 PMC2753066

[B69] Wood K. Nishida S. Sontag E. D. Cluzel P. (2012). Mechanism-independent method for predicting response to multidrug combinations in bacteria. *Proc. Natl. Acad. Sci. U.S.A.* 109 12254–12259. 10.1073/pnas.1201281109 22773816 PMC3409729

[B70] Wood K. (2016). Pairwise interactions and the battle against combinatorics in multidrug therapies. *Proc. Natl. Acad. Sci. U.S.A.* 113 10231–10233. 10.1073/pnas.1612365113 27588905 PMC5027426

[B71] Worthington R. J. Melander C. (2013). Combination approaches to combat multidrug-resistant bacteria. *Trends Biotechnol.* 31 177–184. 10.1016/j.tibtech.2012.12.006 23333434 PMC3594660

[B72] Xu J. Cui K. Shen L. Shi J. Li L. You L. (2019). Crl activates transcription by stabilizing active conformation of the master stress transcription initiation factor. *Elife* 8:e50928. 10.7554/eLife.50928.sa2 31846423 PMC6917491

[B73] Yates A. Akanni W. Amode M. R. Barrell D. Billis K. Carvalho-Silva D. (2016). Ensembl 2016. *Nucleic Acids Res.* 44 D710–D716. 10.1093/nar/gkv1157 26687719 PMC4702834

[B74] Yeh P. Tschumi A. I. Kishony R. (2006). Functional classification of drugs by properties of their pairwise interactions. *Nat. Genet.* 38 489–494. 10.1038/ng1755 16550172

[B75] Young J. W. Elowitz M. B. (2011). Mixed messages: how bacteria resolve conflicting signals. *Mol. Cell* 42 405–406. 10.1016/j.molcel.2011.05.005 21596304 PMC4117311

[B76] Zaslaver A. Bren A. Ronen M. Itzkovitz S. Kikoin I. Shavit S. (2006). A comprehensive library of fluorescent transcriptional reporters for *Escherichia coli*. *Nat. Methods* 3 623–628. 10.1038/nmeth895 16862137

[B77] Zhou A. Kang T. M. Yuan J. Beppler C. Nguyen C. Mao Z. (2015). Synergistic interactions of vancomycin with different antibiotics against *Escherichia coli*: trimethoprim and nitrofurantoin display strong synergies with vancomycin against wild-type *E. coli*. *Antimicrob. Agents Chemother.* 59 276–281. 10.1128/AAC.03502-14 25348521 PMC4291362

