# Supplementary material for: Uncovering Key Metabolic Determinants of the Drug Interactions Between Trimethoprim and Erythromycin in Escherichia coli
Source: Front Microbiol. 2021 Oct 20;12:760017. doi: 10.3389/fmicb.2021.760017 (PMC8564399; doi:10.3389/fmicb.2021.760017)
Supplement: Supplementary Figure 1 — (A) Venn diagram for differentially expressed genes in TMP, ERY and TMP + ERY relative to the no-drug control group. (B) Linear regression of log2 fold-changes for differentially expressed genes that are common to both the TMP and TMP + ERY groups. [file Data_Sheet_1.docx]

Supplementary Material

# Supplementary Figure 1


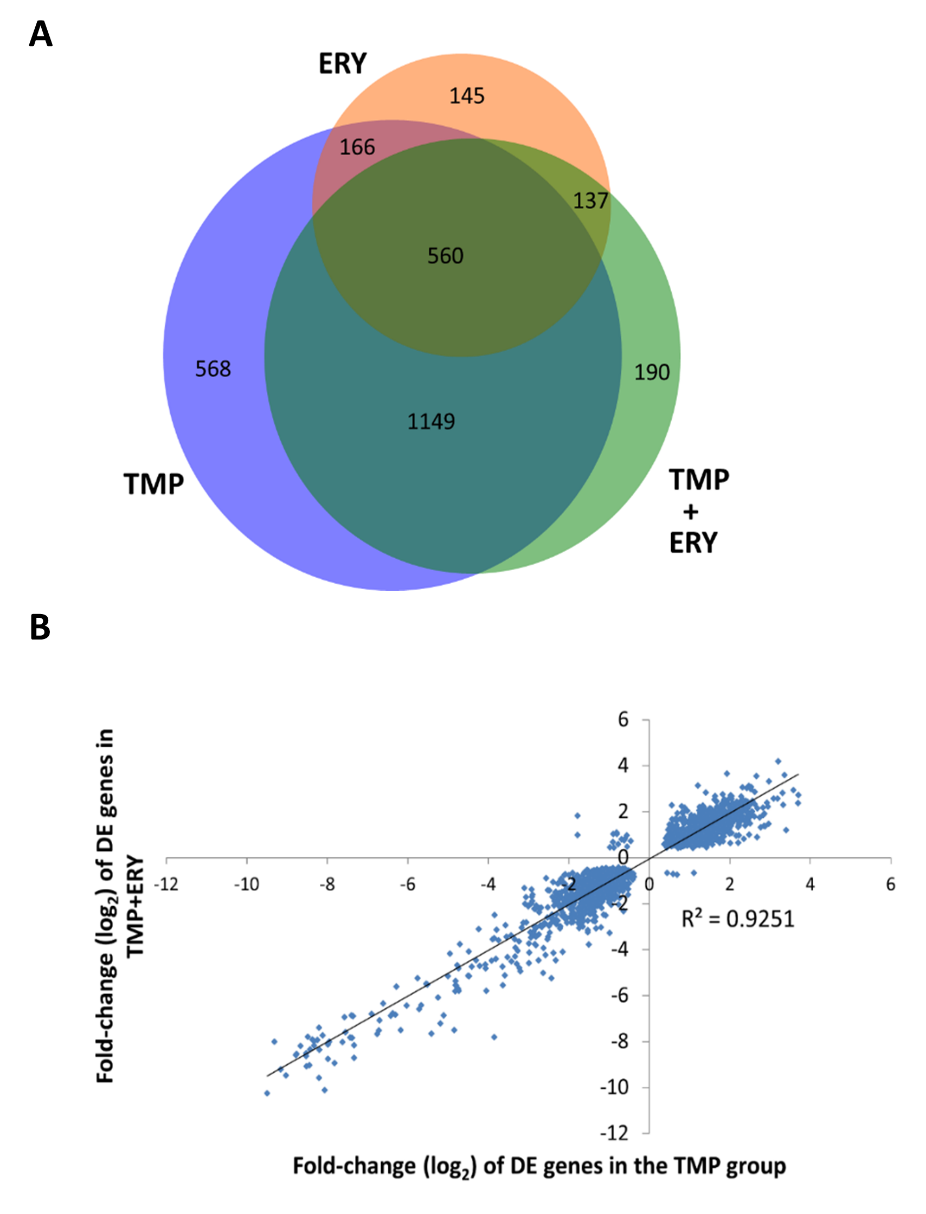


**A. Venn diagram for the number of differentially expressed genes (*P*<0.01 after Benjamini-Hochberg correction).** The areas of the sectors are proportional to the number of genes within those sectors.

**B. Linear regression of log_2_ fold-changes for differentially expressed genes that are common to TMP and TMP+ERY revealed a high degree of similarity in differential gene expression patterns and directions of deviation relative to the no-drug control group.** Each of the 1709 data points represents a gene that is differentially expressed in both groups.

# Supplementary Figure 2


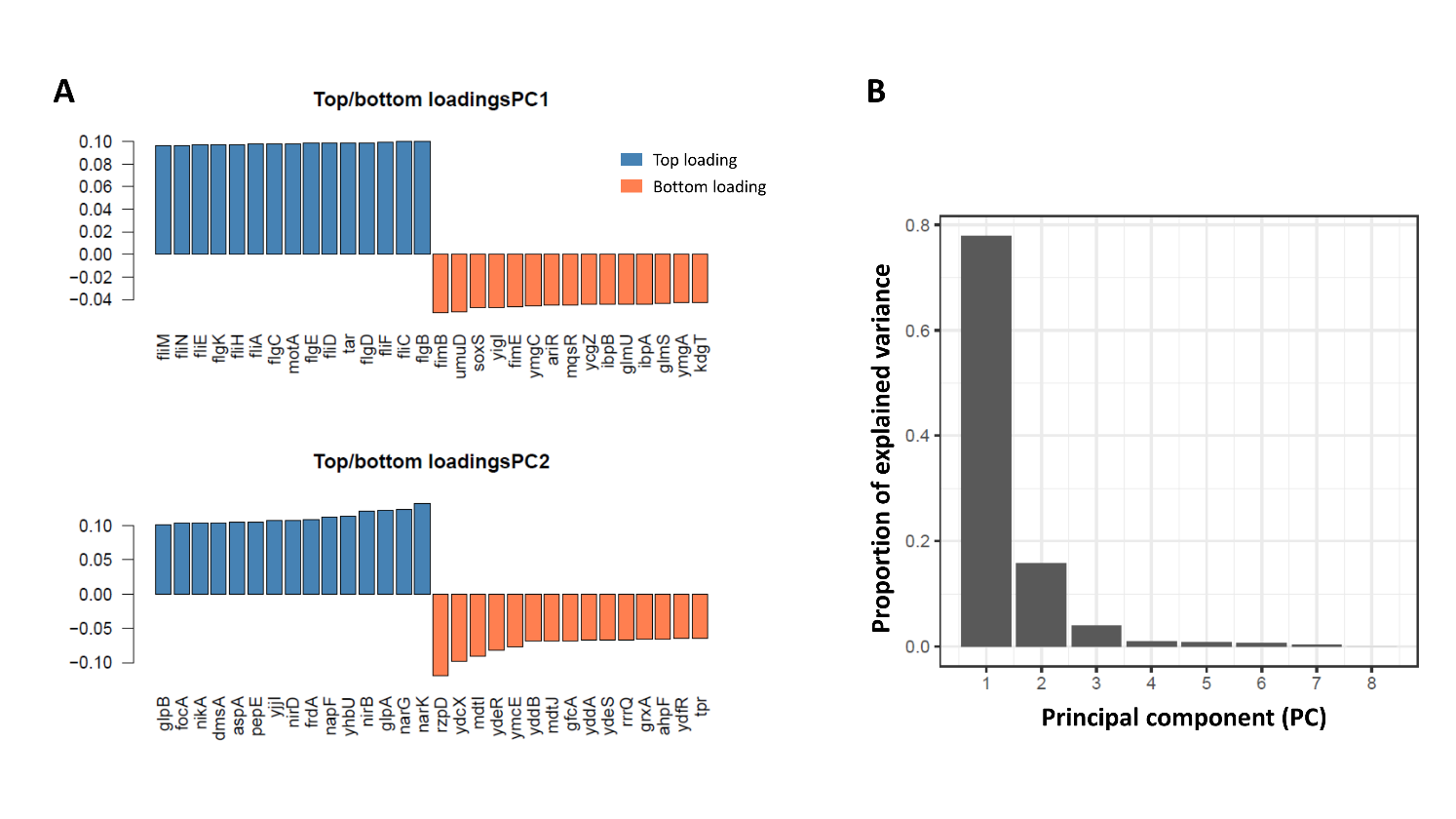


**A. Top and bottom gene loadings on principal components 1 and 2.** The names of the top and bottom 15 genes are shown for PC1 and PC2.

**B. Scree plot for the principal component analysis (PCA).** PC1 and PC2 together explained 94% of the variance in the PCA.

# Supplementary Figure 3


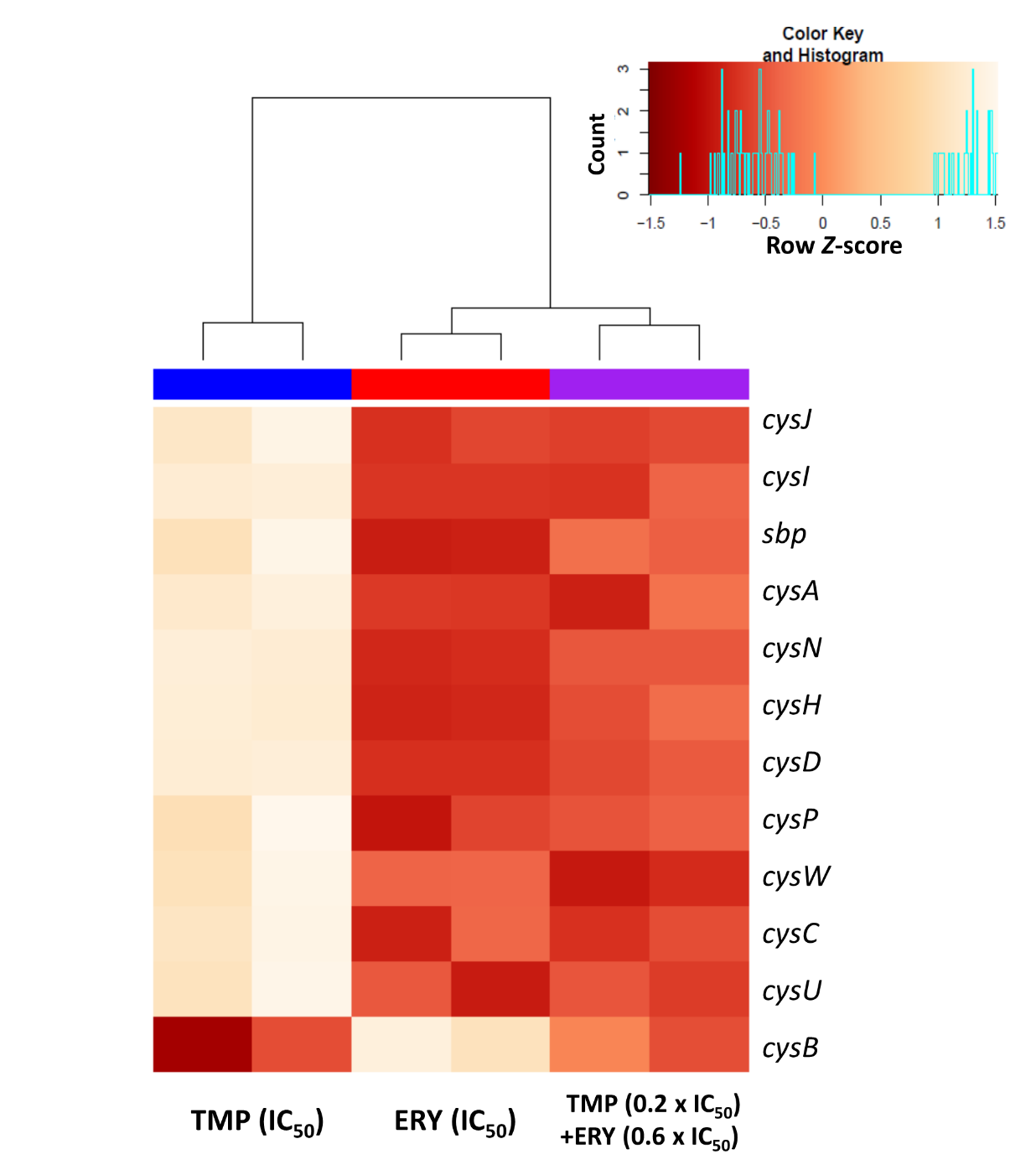


**Two-way hierarchical clustering of *Z*-score transformed gene expression levels of the sulfate reduction pathway for three sample groups with the same 50% reduction in exponential growth rate.** The TMP+ERY group clustered more closely with ERY (IC_50_) than with TMP (IC_50_), which is the same trend as that shown in Figure 3B.

# Supplementary Figure 4


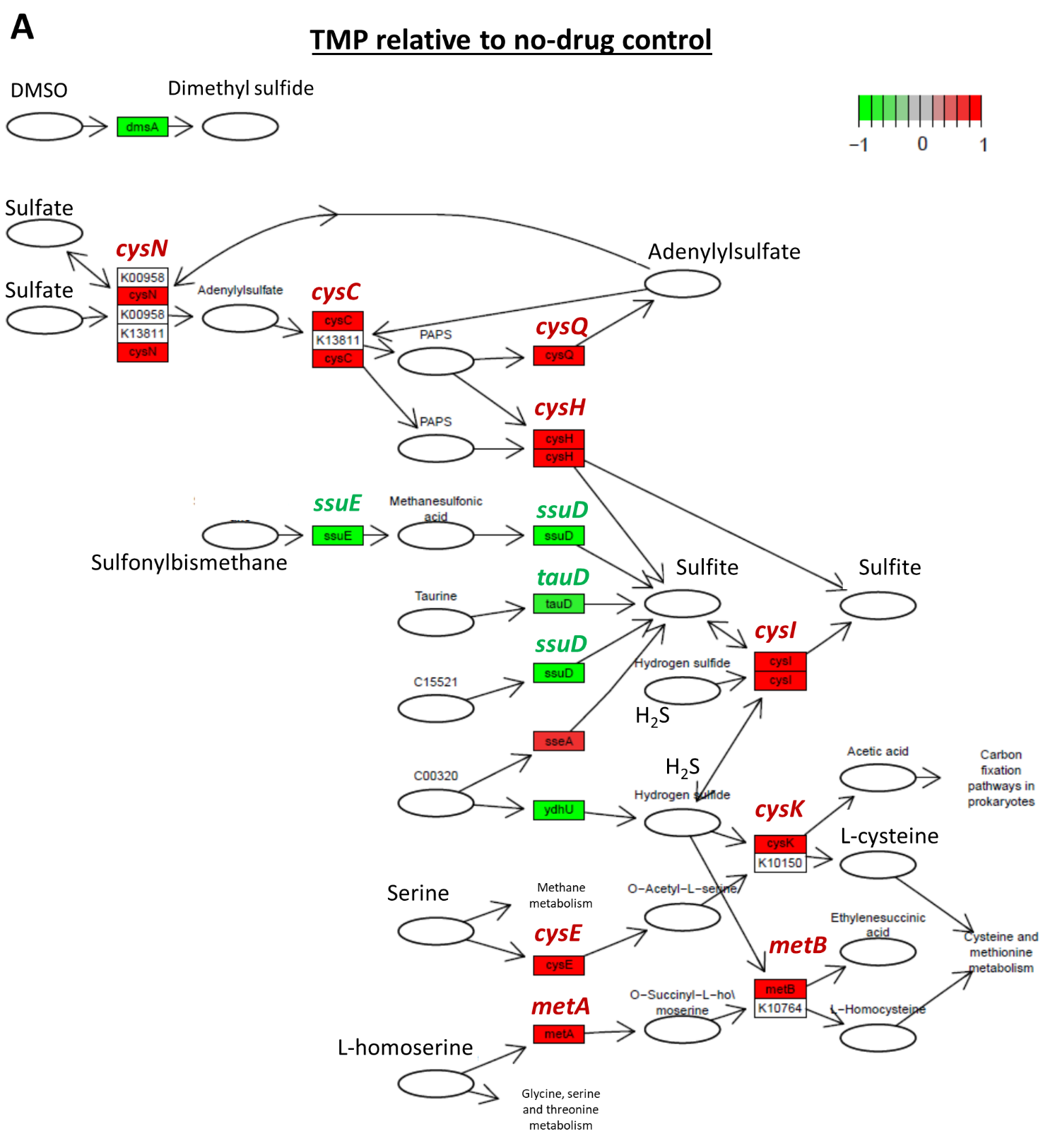


**
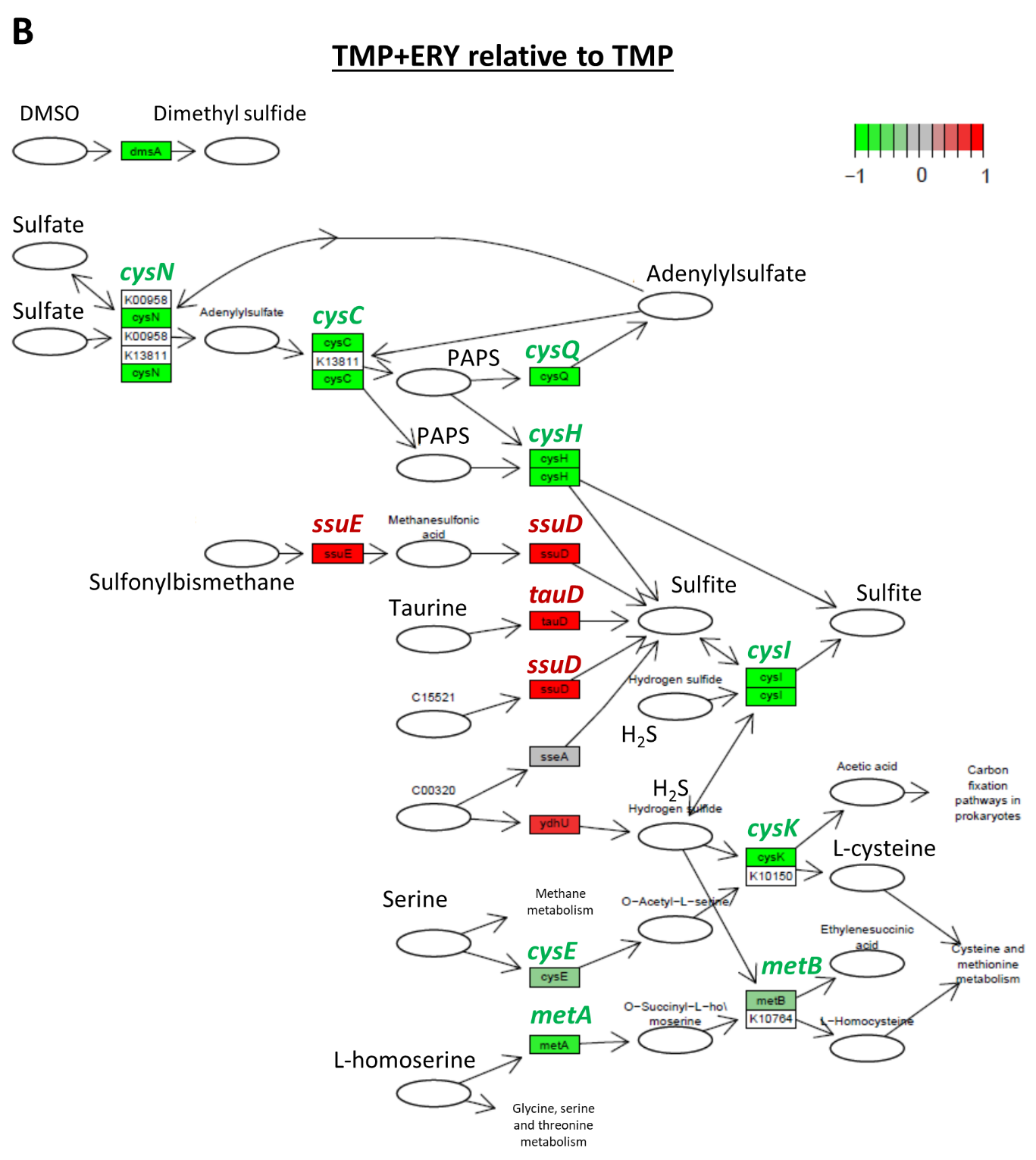
**

**Gene set analysis for the assimilatory sulfate reduction pathway and neighboring nodes for TMP versus no-drug control (A) and TMP+ERY versus TMP (B).** Relative to the TMP group, TMP+ERY showed significant downregulation of the assimilatory sulfate reduction pathway and upregulation of *tauD* and *ssuD/ssuE*, whose gene products utilize alternative sulfur sources under sulfate starvation.

# Supplementary Figure 5


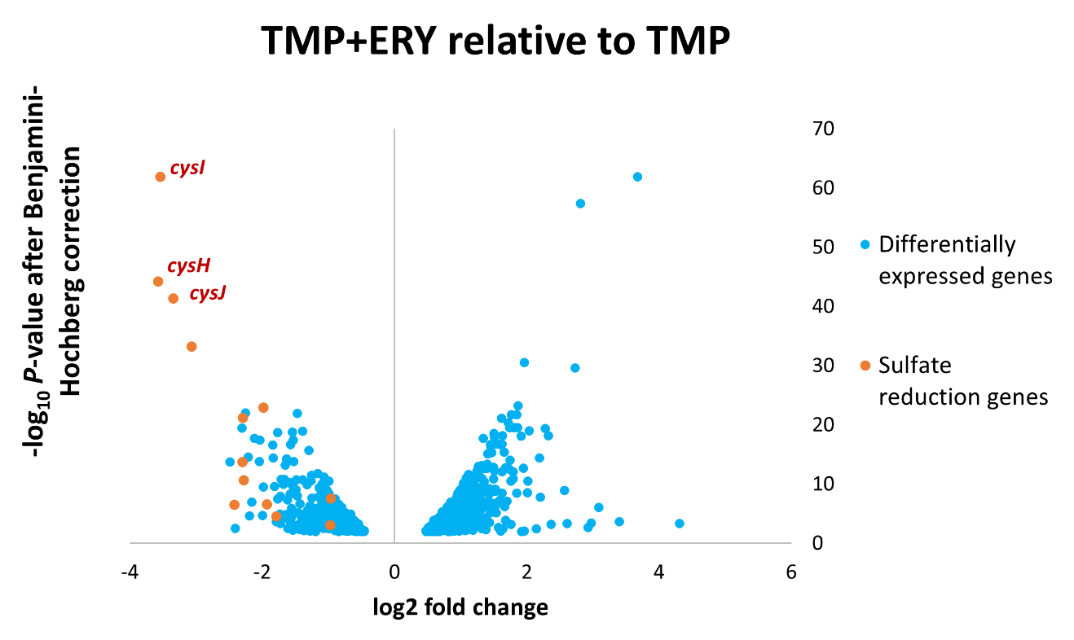


**Volcano plot for the TMP+ERY versus TMP pairwise comparison.** Significantly downregulated genes in this pairwise comparison from the assimilatory sulfate reduction pathway are in orange.

# Supplementary Figure 6


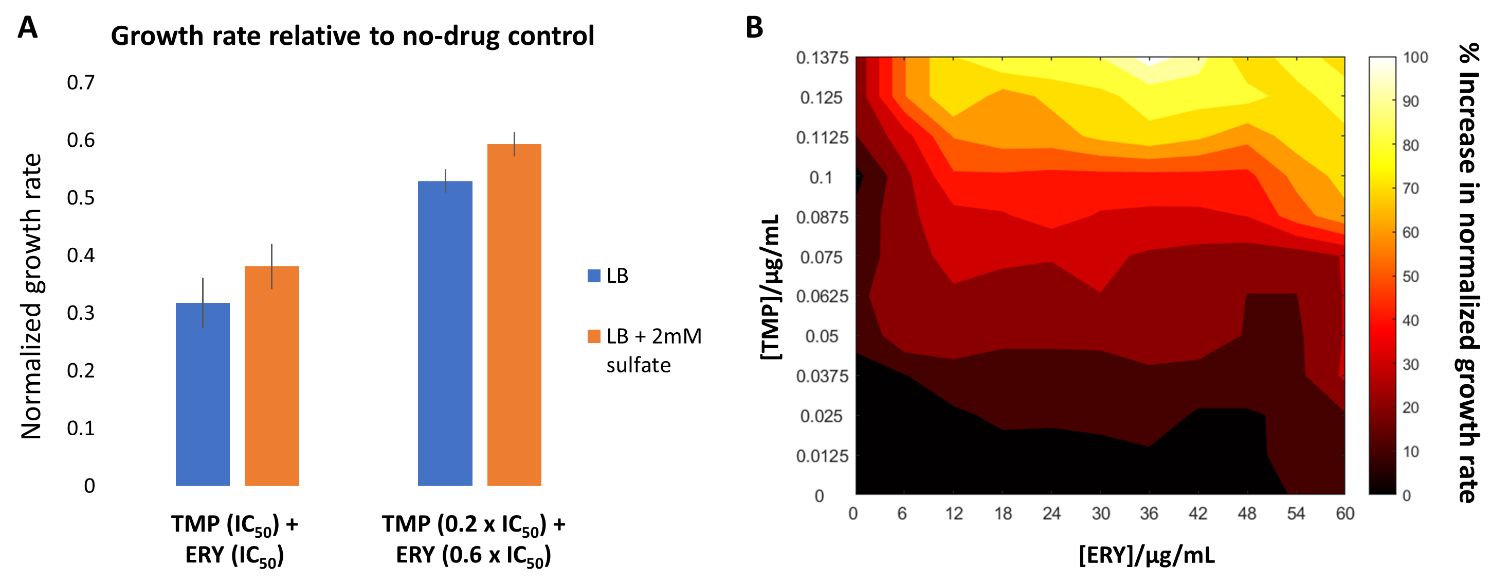


**A. Normalized growth rate relative to the no-drug control for the two combinations of TMP and ERY concentrations.** Supplementation with 2 mM sulfate resulted in increase in average normalized growth rate (n = 3; error bars represent standard error of the mean).

**B. Percentage increase in normalized growth rate with 2 mM sulfate supplementation relative to LB without exogenous sulfate in 2D TMP and ERY concentration space.**

# Supplementary File 1

Genome-wide differential gene expression in five pairwise comparisons: (1) TMP against no-drug control, (2) ERY against no-drug control, (3) TMP+ERY against no-drug control, (4) TMP+ERY against ERY and (5) TMP+ERY against TMP are shown in in the Microsoft Excel file. Differentially expressed genes are displayed in blue.

# Supplementary Table 1

| **File name** | **Descriptions** |
| --- | --- |
| 1-1_s.bam | *E. coli* MG1655 treated with TMP (IC_50_); biological replicate 1 |
| 2-1_s.bam | *E. coli* MG1655 treated with TMP (IC_50_); biological replicate 2 |
| 1-6_s.bam | *E. coli* MG1655 treated with ERY (IC_50_); biological replicate 1 |
| 2-6_s.bam | *E. coli* MG1655 treated with ERY (IC_50_); biological replicate 2 |
| 1-7_s.bam | *E. coli* MG1655 treated with TMP (IC_50_) + ERY (IC_50_); biological replicate 1 |
| 2-7_s.bam | *E. coli* MG1655 treated with TMP (IC_50_) + ERY (IC_50_); biological replicate 2 |
| 1-8_s.bam | *E. coli* MG1655 no-drug control; biological replicate 1 |
| 2-8_s.bam | *E. coli* MG1655 no-drug control; biological replicate 2 |
| MG1655.genome | Gene annotation file |
| MG1655.fa | Reference genome FASTA file |

**Supplementary Table 1**: Accession numbers of BAM and reference genome files for the RNA-Seq transcriptomic dataset in Dryad repository (doi:10.5061/dryad.bk3j9kdcn).
